# Synthesis and Properties of Zinc-Modified Hydroxyapatite

**DOI:** 10.3390/jfb11010010

**Published:** 2020-02-20

**Authors:** Daria Lytkina, Anastasiya Gutsalova, Dmitriy Fedorishin, Natalya Korotchenko, Rafik Akhmedzhanov, Vladimir Kozik, Irina Kurzina

**Affiliations:** 1 Laboratory of Chemical Technology, National Research Tomsk State University, Lenin 36, 634050 Tomsk, Russia; darya-lytkina@yandex.ru (D.L.); strix187@yandex.ru (D.F.); korotch@mail.ru (N.K.); iskander_y@mail.ru (R.A.); vkozik@mail.ru (V.K.); 2 Department of Life Safety and Biomedical Disciplines, Tomsk State Pedagogical University, Kievskaya 60, 634061 Tomsk, Russia

**Keywords:** zinc-modified hydroxyapatite, phase composition, biological activity

## Abstract

Hydroxyapatites modified with metal ions are the main inorganic components of bone tissue and are approved for use as components for biocomposites and coatings for surgical implants. This study examined prototypes of functional materials for bone implants based on hydroxyapatite modified with zinc ions. Zinc-modified hydroxyapatite was composed and synthesized. Using the XRD method, the phase composition was established. Using SEM, EPMA, and low-temperature nitrogen adsorption (BET) methods, surface properties were investigated. Antibacterial activity and biocompatibility have been established. The studied materials have antimicrobial activity; the samples did not cause significant changes in either the internal organs or the general condition of laboratory animals during the entire experiment.

## 1. Introduction

Upon entering the era of personalized medicine, the development of successfully applied materials for implants implies the right compromises regarding the composition of the material, its shape, structure, mechanical characteristics, biocompatibility, proangiogenic or proosteogenic properties and antimicrobial activity. Due to these factors, the development and implementation of new kinds of osteoplastic materials in clinical practice remains a topical issue. An important advantage of these materials over classical materials intended for artificial bone implants is biochemical compatibility, expressed in a lower intensity of inflammatory reactions in the tissues surrounding the implant. The limitation, in terms of autografts and allografts, has forced scientists to develop various synthetic-doped/substituted hydroxyapatites as an alternative [1,2,3,4,5,6].

Hydroxyapatites modified with metal ions are the main inorganic components of bone tissue that are approved for use as components for biocomposites and coatings for surgical implants. These materials are inert to living tissues and have a low toxicity. In addition, due to their applications, the likelihood of allergic reactions, inflammation, and mutagenic effects is quite low. Moreover, modified hydroxyapatites can accelerate the process of reparative osteogenesis at the implantation site and enhance the proliferation of osteoblasts [7]. A wide variety of possibilities for adding cations with different ionic radii is determined by the high “flexibility” of the lattice and the good structural stability of hydroxyapatite (HA) [1,8,9]. Artificially substituted HAs apparently have a number of significant advantages over stoichiometric HAs (for example, changes in lattice constants and the unit cell volume, formation of defects, surface charge distribution) and morphological modifications by cations [10,11,12,13,14,15,16,17,18].

In this regard, it seems particularly relevant to conduct research on the study of biocompatibility of a series of modified hydroxyapatites, which are structural components for biocompatible bone implants [11].

When a biomaterial is implanted into the body, reactions occur at the interface between the tissue and the material, including acute inflammation, wound healing, and the body’s reaction to a foreign body. In this case, acute inflammation is necessary for healing wounds and restoring homeostasis at the site of injury. However, excessive pro-inflammatory properties of the material can cause various pathological reactions, which ultimately lead to the complete rejection of the implant.

This research examined prototypes of functional materials intended for bone implants based on hydroxyapatite modified with zinc ions. Zinc is one of the most common trace bone cations and is a cofactor of hundreds of enzymes involved in bone metabolism. Hydroxyapatite modified with zinc ions increases the viability of osteoblast cells, their adhesion, proliferation and differentiation; it also stimulates osteogenic activity, bone growth and healing in case of damage [19]. 

HA doped with zinc has been confirmed as an effective antimicrobial agent against gram-positive and gram-negative bacteria that are often found at the implantation site: for example, S. aureus, Streptococcus mutans, Staphylococcus epidermidis, Enterobacter aerogenes, *E. coli* [2,20,21,22,23,24,25]. The release of Zn^2+^ ions acted against fungal infection, and after 72 h the C. albicans biofilm decreased significantly, with a zinc content of 3% [26]. In the absence of light, with a lower content of Zn^2+^ ions (~1%), the number of C. albicans cells also decreased markedly [27]. Doping with zinc has a positive effect on the inhibition of the formation of bacterial plaques on enamels and on the improvement of enamel remineralization during dental prosthetics. However, at high concentrations of ZnHA (2%), biocompatibility was impaired, although it was effective against enamel bacteria (S. mutans, Lactobacillaceae and Streptococcus sobrinus), while ZnHA (1%) increased both the proliferation of osteoblasts and their antibacterial properties. The vast majority of studies [20,22,23,26,27,28] of zinc-substituted HA indicate zinc content in the concentration range 0.1–4%. The best results in terms of biocompatibility, osteoconductivity and antimicrobial activity are achieved at a Zn^2+^ content of ~1–2% [20,21,24]. It is noteworthy that in vivo tests on model animals showed the ability of ZnHA to accelerate the formation of new bone compared with pure HA during implantation in rats [29] and rabbits [30] for one and two months, respectively. In this regard, it seems very relevant to conduct a comprehensive chemical study of Zn-modified hydroxyapatites, as well as to study their biocompatibility and antibacterial properties, depending on the zinc concentration.

## 2. Results and Their Discussion

**Figure 1:**
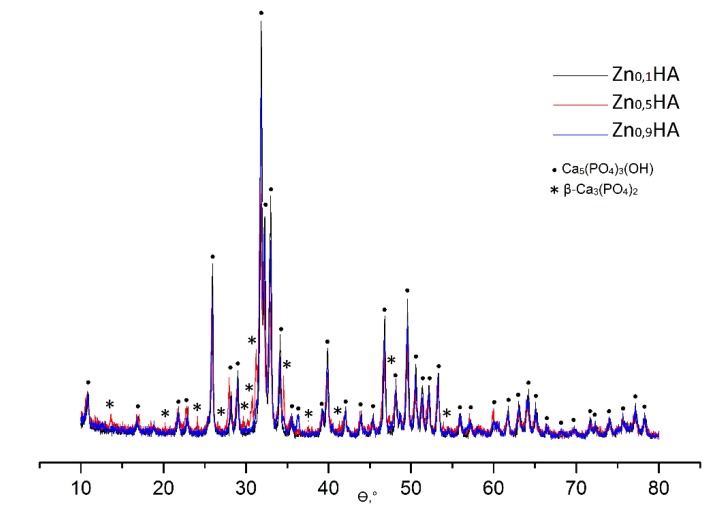
X-ray patterns of samples Zn_0.1_HA, Zn_0.5_HA, Zn_0.9_HA.

The qualitative composition of HA and ZnHA samples was determined by XRD methods. Diffractograms of synthetic HA (Figure 1) are typical of the apatite structure. The appearance of the diffractograms of the samples under study indicates a high degree of crystallinity. According to the XRD data, the crystalline phase of samples Zn_0.1_HA, Zn_0.5_HA, Zn_0.9_HA corresponds to HA with a unit cell of Ca_5_(PO_4_)_3_OHhex. It was shown that the main phase of the samples is hydroxyapatite Ca_5_(PO_4_)_3_(OH). The main product of Zn_0.5_HA is HA; in addition, there is ~14.5% of the impurity phase β-Ca_3_(PO_4_)_2_ (Table 1).

Peaks of extraneous phases (CaCO_3_, CaO and Ca_3_(PO_4_)_2_) are absent, which indicates the formation of stoichiometric HA during synthesis. The diffractograms of natural apatites and synthetic HA coincide. According to the XDR data, the samples can be considered as an apatite of hexagonal syngony. The unit cell parameters of all the samples are in satisfactory conformity with the data of the International Center for Diffraction Standards (according to JCPDS No. 9-432: a = 9.418 Å; c = 6.884 Å [31]).

The elemental composition of the synthesized ZnHA powders according to the results of EMPA shows the molar ratio of elements (Ca + Zn)/P is 1.4–1.9 (Table 2), which correspond to the ratio of elements Ca/P for the bone tissue (1.4–2.0) [31].

**Figure 2:**
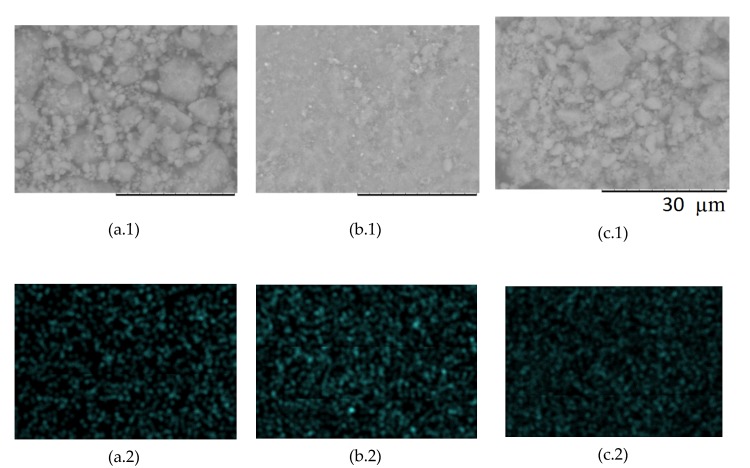
(**1**) Micrographs of the surface, (**2**) Distribution of Zn over the surface of the calcined samples: a—Zn_0.1_HA, b—Zn_0.5_HA, c—Zn_0.9_HA.

Figure 2(a.1–c.1) shows the image of the samples at the maximum possible magnification, where the presence of agglomerates of various shapes and sizes is visible. Due to the different phase composition of the sample Zn_0.5_HA, the appearance of its surface also undergoes a change: the particle size of the biphasic product is much smaller than single-phase products. Electron microprobe analysis (EMPA) shows (Figure 2(a.2–c.2)) that the distribution of zinc ions over the surface of the modified HA samples (Figure 2(a.1–c.1)) is homogeneous, and there are no local inhomogeneous zones.

**Figure 3:**
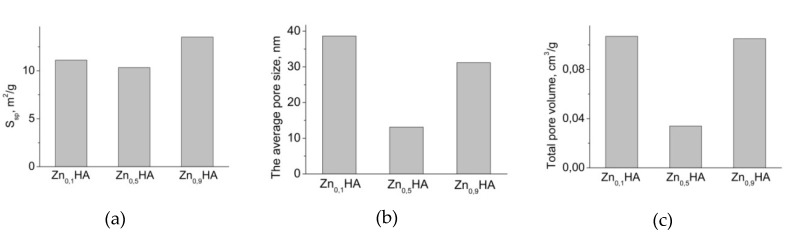
(**a**) specific surface area Δ ± 10%; (**b**) average pore size; (**c**) total pore volume of samples Zn_0.1_HA, Zn_0.5_HA, Zn_0.9_HA.

The results of low-temperature nitrogen adsorption (Figure 3) show that the specific surface areas of the samples are similar in value. However, the total and average pore volume of the ZnHA sample (x = 0.5) is considerably smaller than that of other materials. This can be attributed to the phase composition of this sample, which is biphase, in contrast to other monophase samples. This shows that even a small fraction (~14.5%) of β-Ca_3_(PO_4_)_2_ formed during crystallization can significantly affect the relief, which becomes flatter (Figure 2, b1), and the structure of the porosity of the materials. 

The studied samples of the ZnHA series showed the antibacterial activity of various intensities (Table 3). Moreover, the ZnHA sample (x = 0.1) did not have an inhibiting influence on the number of Escherichia coli. The number of bacteria in a liquid medium with this sample is less than that in the control sample, but no significant differences were statistically established (p > 0.05).

ZnHA samples (x = 0.5 and 0.9) showed antibacterial activity, having decreased the number of Escherichia coli in comparison with that of the control sample. The ZnHA sample (x = 0.9) showed the highest antibacterial activity among all the samples presented in the study, having reduced the number of bacteria by almost an order of magnitude. This was statistically confirmed (p < 0.001). 

As can be seen from Table 4, unmodified hydroxyapatite also has antimicrobial activity (p < 0.05). Its antimicrobial activity and cytotoxicity in vivo studies is approximately equal to the ZnHA sample (x = 0.1). Therefore, it can be concluded that the ZnHA sample is approximately equal to unmodified hydroxyapatite in its biological effects.

To confirm the differences between the samples, the T-test was used. The normality of the distribution was confirmed using the Shapiro–Wilk test.

It can also be noted that the modification of hydroxyapatites with metal ions is apparently key to achieving potent antimicrobial activity. Such materials can even withstand highly resistant microorganisms adapted to conventional antibiotics. In this case, the toxic side effects of such materials will be significantly limited. The simultaneous release of ions with different mechanisms of action can not only prevent the adaptation of bacteria and fungi, but also expand the antimicrobial spectrum of action against a larger number of strains of pathogenic microorganisms.

As a result of testing the samples on the laboratory animals, the following data were obtained. Throughout the experiment with the animals, they did not show any deviations from the natural dynamics of the body weight. Water and feed intake remained at the ordinary level for the animals of this age. No changes in their behavior were detected. There were no cases of unplanned death of animals during the entire experiment. No damages to the surgical suture were found in all the animals.

As a result of necropsy of the experimental animals, it was found that all implants, to one degree or another, negatively influenced the surrounding tissues. In several cases, the implants were not removed. This is due to an acute necrotic reaction of the body to the implant. At the same time, one-sided perforation of the skin with an implant prolapse was observed. In this case, hyperemia of the surrounding tissues and exudate were detected in the peri-implant space. Hemorrhages were also detected around the implants. In the peri-implant space in the area of perforation of the skin, it was possible to identify an area of necrosis with a diameter of 3–4 mm and purulent exudate. No fat infiltrate was found at the implant sites, and fibrous capsules around the implants were poorly expressed. However, it should be noted that this type of reaction was observed only in several cases, and their presence can be explained by the local immune response of a particular body.

After extraction, the implants were air-dried at room temperature and weighed. The average mass of implants before the start of the experiment was 170–200 mg and, after the end of the experiment, 175–210 mg (p > 0.05). The change in the mass of the implants is associated with the ingrowth of fibrous tissue in their surface.

The ZnHA group implants (x = 0.9) showed a local rejection reaction, but inflammation rather than necrosis predominated. Hemorrhages were detected at the implant sites, but in these cases the implants were in a pronounced fibrous capsule. At the installation site there was a slight fatty infiltrate. No exudate was found at the installation sites. In two cases, perforation of the skin at the site of implant installation was observed: in the first case bilateral, in the other, unilateral. At the same time, the area of pronounced necrosis was slightly smaller (approximately 1 mm). In the case of perforation, purulent exudate was observed in all cases.

The ZnHA group implants (x = 0.5) showed the whole complex of perimplantation reactions of the surrounding tissues, but without causing necrosis and perforation of the skin. When studying the ZnHA group (x = 0.1), unilateral perforation of the skin was observed in two animals. In other cases, the same reactions were observed as in the previous group of zinc-modified HA. In all ZnHA groups, the implants maintained structural integrity throughout the experiment; no fragmentation was observed.

The implants of the positive control group caused the entire spectrum of reactions typical of that of the ZnHA groups. Unilateral perforation of the skin was observed in two animals. In the negative control group, no traces of pathological processes were found in the areas of pockets formation.

As a result of the conducted experiment, it was found that all the materials presented in the research produced a local rejection reaction, and the severity of this reaction could vary. Moreover, complete perforation of the skin was observed only in five cases out of 30. In the positive control group, perforation of the skin was observed in two cases. Moreover, previous studies have shown some antibacterial activity and cytotoxicity of these materials. It should be noted that the reaction of the local tissues to all the samples of the ZnHA group as a whole was inflammatory in nature, but not necrotic. This is confirmed by the presence of an intensive vascularization process and formation of pronounced fibrous capsules at the sites of installation of these implants of this group. The local response to the implants of the positive control group was similar to that of the ZnHA group. No differences between them were found, which indicates similar levels of cytotoxicity of these materials.

It should be especially noted that, in all the experimental animals during the autopsy, there were no traces of systemic toxic organ damage. Based on this, it can be noted that the acute reaction of rejection of implants, even when necrotic, is predominantly local and does not cause systemic damage to the body. This may be evidence of the fact that a toxic concentration of zinc has not been reached. Low hemodiffusion at the implant sites and the low solubility of HA in water have contributed to that.

The implants of the positive control group caused the entire spectrum of reactions typical of ZnHA groups. Unilateral perforation of the skin was observed in two animals. In the group of negative control in the areas of pockets formation, no traces of pathological processes were visually detected.

**Figure 4:**
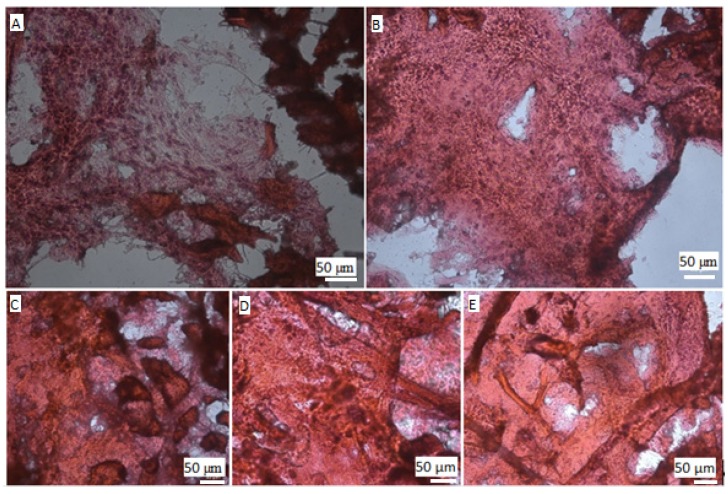
Micrographs of tissue samples of the experimental animals taken from implant sites of various groups. (**A**)—control; (**B**)—unmodified hydroxyapatite; (**C**)—Zn_0.1_HA; (**D**)—Zn_0.5_HA; (**E**)—Zn_0.9_HA. The fibrous tissue in the experimental groups is clearly visible. The implants were installed contralaterally in the right and left axillary folds. The scale is 50 µm.

The histological examination showed that no significant changes were found in the negative control group. Identified traces of fibrosis were the result of the mechanical tissue damage during surgery (Figure 4A). The positive control group had a similar result, but the tissues at the implant site were slightly denser (Figure 4B). In all the ZnHA groups at the installation sites, the connective tissue with a large number of collagen fibers was found (Figure 4C–E). This connective tissue surrounded the implant with a continuous capsule. This is evidence of a local implant rejection process. This reaction was observed in all the groups, and its intensity did not differ. However, a slightly larger number of collagen fibers in the ZnHA group (x = 0.9) should be noted (Figure 4E). This indicates a slightly greater toxicity of this sample compared to that of others. It can also be noted that the micrograph of the sample of the ZnHA group (x = 0.5) is somewhat different from other groups. This may be due to the different density of fibrous tissue and the different configuration of collagen fibers at the implant site.

As a result of the conducted experiment, it was found that all the materials presented in the research caused a local rejection reaction, and the severity of this reaction could vary. Moreover, complete perforation of the skin was observed only in five cases out of 30. In the positive control group, perforation of the skin was observed in two cases. It should be noted that, as a whole, the reaction of the local tissues to all the samples of the ZnHA group was inflammatory in nature, but not necrotic. This is confirmed by the presence of an intensive vascularization process and the formation of pronounced fibrous capsules at the sites of installation of these implants in this group. The local response to the implants of the positive control group was similar to that of the ZnHA group. No differences were found between them, which indicates similar levels of cytotoxicity of these materials. This has also been histologically confirmed.

It should be noted that there was a relationship between the concentration of zinc in the sample and its toxic effects. Samples with a high content of zinc ions caused generally greater toxic effects than samples with a low content of zinc.

Resorption of the implants when the experimental animals were in the body during this period was not found. The microscopy of implants revealed the presence of ingrown connective tissue in all samples. The presence of this tissue could compensate for the change in mass. A slight change in mass may be due to a slight release of ions from the implants. Low hemodiffusion at the site of installation could also be the reason for the lack of resorption of implants. 

After sample extraction, EPMA was also performed (Table 5). During the experiment, the concentration of zinc ions on the surface is significantly reduced by ~20–40%, and the higher the zinc yield, the higher the lower its initial concentration.

It should be especially noted that, during the autopsy, there were no traces of systemic toxic damage to organs in all the experimental animals. Based on this, it can be noted that the acute reaction of rejection of implants, even a necrotic one, is predominantly local and does not cause systemic damage to the body. This is also confirmed by the EPMA results. The yield of zinc ions from the implants is negligible, therefore the toxic effects are purely local. This may be evidence of the fact that a toxic concentration of zinc has not been reached. The low hemodiffusion at the implant sites and low solubility of HA in water contribute to this.

## 3. Materials and Methods

### 3.1. Sample Synthesis

To obtain samples of HA, ZnHA and to establish physicochemical properties depending on the degree of Zn2+ ion content, a liquid-phase microwave synthesis of samples with different contents of these ions was carried out according to Equation (1)

10Ca(NO_3_)_2_ + 6(NH_4_)_2_HPO_4_ + 8NH_4_OH → Ca_10_(PO_4_)_6_(OH)_2_ + 20NH_4_NO_3_ + 6H_2_O (1)

To obtain 10 g of HA in a beaker, freshly prepared solutions of calcium nitrate (Cm = 0.5 mol/L) and ammonium hydrogen phosphate (C_m_ = 0.3 mol/L) are mixed with stirring in 200mL, required to obtain the ratio of elements Ca/P = 5/3, characteristic of hydroxyapatite. By adding a concentrated (25%, ⍴ = 0.9 g/mL) ammonia solution, the pH in the solution is adjusted to 10–11. The reaction mixture is subjected to microwave radiation at a power of 110 W and a temperature of ~100 °C for 35–40 min. Then, the precipitate formed is settled in the mother liquor at room temperature for 48 h. The precipitate is filtered off, and then left to dry in an oven (~20 h) at a temperature of 90 °C. Next, the dried precipitate is ground in a mill and calcined in a muffle furnace at 800–900 °C for 2–4 h. When carrying out synthesis of HA by this method, the product yield is 92.0–94.0%. The synthesis of ZnHA powders is carried out by analogy.

The scheme for obtaining samples is shown in Equation (2)

(10 − x)Ca(NO_3_)_2_ + 6(NH_4_)_2_HPO_4_ + xZn(NO_3_)_2_ + 8NH_4_OH → Ca_10-x_Zn_x_(PO_4_)_6_(OH)_2_ + 6H_2_O + 20NH_4_NO_3_ (2)

where x (mol) = 0.1; 0.5; 0.9.

The calculated amount of dry zinc nitrate was dissolved in a pre-prepared solution of calcium nitrate. Next, the synthesis was carried out according to the selected conditions, as previously presented. All samples were ground in a mill. Powders are white substances. Before the studies, all samples were calcined for 2 h at 800 °C.

The study of the microstructure of samples is often accompanied by X-ray microanalysis (EPMA). A characteristic feature of the method is its locality—the maximum excitation region is 1 μm. The analysis was performed on a Hitachi TM3000 scanning electron microscope (Hitachi, Tokyo, Japan). The specific surface area (Ssp), the volume and pore size of ZnHA-based powders were measured by adsorption followed by degassing at a pressure of ~0.1 Pa at 200 °C for two hours on a TriStar 3020 gas adsorption analyzer (using the BET method) (relative error ∆ ± 10%). The analysis was performed on an XRD-6000 diffractometer (Shimadzu, Tokyo, Japan) according to the powder method. Analysis conditions: voltage 40 kV, speed 5 degrees/min.

### 3.2. Study of Biological Activity of Samples

#### 3.2.1. Study of Antibacterial Activity of Samples

The antibacterial activity of three samples of hydroxyapatite (with different molar metal contents (x = 0.1; 0.5; 0.9)) was determined by Koch method for insoluble compounds in a powder form. Escherichia coli American Type Culture Collection (ATCC) 25,922 were used as a test object. The bacterial culture growth on the standard Lysogeny broth (LB) medium was used as a control object. The medium for the cultivation of Escherichia coli was standard for this bacteria–LB medium. Incubation was carried out in a thermostat at a temperature of 37–38 °C. Bacterial inoculation on the standard LB medium containing no samples was used as a control. Each sample was taken in the amount of 0.8 g and placed in a flask with a liquid nutrient medium, LB. Then it was sterilized in the autoclave at 1 ATMG, 120 °C for 30 min. After sterilization, the experiment was performed according to the following scheme:

1. Growth of the stock culture of bacteria. After 24 h, seeding was performed on solid LB nutrient media to establish the initial number of bacteria;

2. A total of 1 mL of the liquid culture of bacteria was transferred into a flask with the test sample. Cultivation of bacteria on the standard LB medium, without adding samples, was used as control;

3. Incubation was carried out for 24 h at a temperature of +37–38 °C;

4. After 24 h, the samples were seeded on a solid LB nutrient media. Each flask was seeded using three dilutions into four Petri dishes with the dense nutrient medium. The dishes were incubated in the thermostat for 24 h at a temperature of +37–38 °C;

5. After 24 h, the number of grown bacterial colonies on the solid nutrient medium in Petri dishes was recorded;

6. The number of the colonies of forming units (CFU) in 1 mL of the culture in the control and with the samples was calculated; the confidence intervals for a 95% significance level were calculated.

#### 3.2.2. Study of the Biocompatibility of Samples in vivo

Each implantable sample was made, cleaned and sterilized in accordance with the technology used for bone implants. All manipulations with implants were performed under aseptic conditions to prevent damages or contamination of the samples before or during implantation.

The HA samples provided for the study were compressed tablets with a diameter of 4 mm and a height of 3 mm. To assess the resorbability of the study objects in the bodies of experimental animals, all samples were weighed immediately before implantation, as well as after the animals were removed from the experiment. The average weight of the HA tablets before the start of the experiment was 170–200 mg. House mice (Mus musculus) aged 4 weeks old and an average weight of 25–30 g (n = 40) served as the test system for the experiment. All the mice were divided into five equal groups: experience, positive control and negative control. The size of each group was five animals. The mice included in the experimental groups were fitted with implants made from modified HA, presented during the study. The mice from the positive control group had implants made of HA not modified with metal ions. The same experimental procedures were performed on the animals from the negative control group and on the animals from other groups. The main difference between this group and other groups was that the animals from this group did not have implants. This group is necessary for the most complete differentiation of the observed effects caused by surgical procedures from the effects caused by the action of implants.

The determination of the biocompatibility of the samples in vivo was as follows. A 5–7 mm long skin incision was made in the sternum of the fixed animal. After that, using a blunt-tip probe, pockets were formed under the skin of the animal, corresponding in size to the size of the implant. A sample in the form of a tablet was placed in this pocket so that the sample was in the axillary fold of the experimental animal. Then, the skin incision was sutured with cotton thread using a surgical needle with a cutting profile. The number of seams corresponded to the length of the incision. The pockets with cylinders installed in them were located contralaterally in the right and left axillary folds. The implants were installed in these places to prevent damages to the sutures and the implants themselves by other animals of this group.

During the experimental manipulations, the animals were under mild zoetilic anaesthetic. Anaesthetic effect was verified by the disappearance of the reaction to pain stimuli (paw prick) and inhibition of the corneal reflex. The anesthetized animal was fixed on the table with its back down.

The duration of the experiment was 45 days. Such a duration allows for the optimal assessment of HA biocompatibility and the difference between effects caused directly by the operation, and those caused by the action of the objects of study. Once every two days, all the experimental animals were examined in a natural habitat to determine pain and general condition. In addition, the mass of the experimental animals, and the consumption of water and feed were measured.

After removing the animals from the experiment, they underwent humane euthanasia followed by complete necropsy. The implants were excised with enough of the surrounding tissue to evaluate the local tissue response. After extraction, the implants were air-dried at room temperature and weighed. Tissue samples were also excised from implant placement sites for further histological examination. Tissue samples of the experimental animals, taken from implant sites, were fixed using a buffered formalin solution according to the standard protocol. After fixation, the tissue samples were frozen and cut using the Microm HM 525 cryotome (Thermo Scientific, Walldorf, Germany) at −22 °C. The thickness of the sections was 50 μm. After cutting, the samples were subjected to hematoxylin-eosin staining according to the standard protocol. The microscope observation of the tissue samples was carried out using a microscope Zeiss AxioImager Z2. Statistical processing of the results was carried out using Microsoft Office Excel and StatSoft STATISTICA v.10.

#### 3.2.3. Keeping, Care and Using of Laboratory Animals

The animals were kept and the experiments were carried out in accordance with the local and international rules—“GOST 33216-2014 Guide for the maintenance and care of laboratory animals. Rules for the maintenance and care of laboratory rodents and rabbits” [32] and “Guide for the Care and Use of Laboratory Animals, 8th edition” [33]. The experimental animals were kept in accordance with the specified regulations in a specially equipped vivarium with free access to water and food, as well as a 12/12 light regime. 

After removal from the experiment, the animals were subjected to humane euthanasia in accordance with international rules. Euthanasia was carried out by cervical dislocation. All the experimental manipulations with animals did not include painful and inflicting suffering procedures and were approved by the Tomsk State University Bioethics Committee.

## 4. Conclusions

All the obtained samples are represented by the hydroxyapatite phase Ca_5_(PO_4_)_3_(OH). Zinc is evenly distributed over the surface, which is confirmed by the EMPA data. However, at a certain molar concentration of zinc Zn_0.5_HA during crystallization, an additional phase, β-Ca_3_(PO_4_)_2_, forms. The formation of the β-Ca_3_(PO_4_)_2_ phase significantly influences the surface characteristics of the samples; in particular, there is a significant reduction in the average size and total pore volume with regard to monophase products.

Biological testing of the samples showed that the modified hydroxyapatite zinc ions have antimicrobial activity, which can reduce or prevent the development of concomitant infections when used as components for bone implants. Despite some cytotoxicity found in in vivo studies, these samples did not cause significant changes in either the internal organs or the general condition of laboratory animals during the entire experiment. All the identified cytotoxic effects were strictly local. There were no general negative reactions of the body to the implants.

## Figures and Tables

**Table 1:** Results of x-ray phase and x-ray diffraction analysis of powders HA, ZnHA.

Sample	Phase	Coherent Scattering Region (nm)
HA	Ca_5_(PO_4_)_3_(OH) (hydroxylapatite)	48
Zn_0.1_HA	Ca_5_(PO_4_)_3_(OH) (hydroxylapatite)	77
Zn_0.5_HA	Ca_5_(PO_4_)_3_(OH) (hydroxylapatite)β-Ca_3_(PO_4_)_2_ (14,5%)	59
Zn_0.9_HA	Ca_5_(PO_4_)_3_(OH) (hydroxylapatite)	63

**Table 2:** Elemental composition of powders ZnHA.

**Sample**	**Content of Elements (at.%)**				
	**O**	**Ca**	**P**	**Zn**	$\frac{Ca\;+\;Zn}{P}$
x (Zn^2+^, mol) is introduced					
0.1	67.47	18.76	13.69	0.18	1.38
0.5	67.84	18.57	12.99	0.72	1.64
0.9	70.94	18.08	9.93	1.06	1.93

**Table 3:** Research results of antimicrobial activity of samples.

No.	Variant	Number, Colony Forming Unit (CFU)/mL
1	Control	(3.96 ± 0.62) × 10^7^
2	Zn_0.1_HA	(3.40 ± 0.30) × 10^7^
3	Zn_0.5_HA	(2.15 ± 0.28) × 10^7^
4	Zn_0.9_HA	(8.31 ± 0.91) × 10^6^

**Table 4:** Research results of antimicrobial activity unmodified hydroxyapatite.

No.	Variant	Number, CFU/mL
1	Control	(1.98 ± 0.91) × 10^10^
2	HA	(1.01 ± 0.49) × 10^10^

**Table 5:** Elemental composition of powders ZnHA before and after in vivo

Sample x (Zn^2+^, mol) is Introduced	Content of Elements (at.%)				
	O	Ca	P	Zn	$\frac{Ca\;+\;Zn}{P}$
**Before in vivo**					
0.1	67.47	18.76	13.69	0.18	1.38
0.5	67.84	18.57	12.99	0.72	1.64
0.9	70.94	18.08	9.93	1.06	1.93
**After in vivo**					
0.1	64.90	22.59	12.15	0.12	1.86
0.5	64.58	22.87	12.17	0.36	1.90
0.9	65.04	21.70	12.41	0.83	1.81
